# Abnormal regional myocardial morphology in patients with left ventricular pressure overload and preserved ejection fraction detected by multiparametric MR tissue mapping

**DOI:** 10.1186/1532-429X-18-S1-P336

**Published:** 2016-01-27

**Authors:** Florian von Knobelsdorff-Brenkenhoff, Anna-Katharina Mueller, Marcel Prothmann, Pierre Hennig, Matthias A Dieringer, Luisa M Schmacht, Andreas Greiser, Jeanette Schulz-Menger

**Affiliations:** 1 Charité Medical Faculty and HELIOS clinics, Working group Cardiovascular MRI, Berlin, Germany; 2 Siemens Healthcare, Erlangen, Germany

## Background

Abnormal myocardial morphology of the left ventricle (LV) may be relevant in patients with LV pressure overload for developing heart failure despite having preserved ejection fraction. We aimed to detect subclinical myocardial tissue changes in patients with aortic stenosis (AS) and hypertensive heart disease (HYP) by quantitative cardiovascular magnetic resonance.

## Methods

Forty-eight patients with pressure overload (33 AS, 15 HYP and LV septum thickness ≥13 mm) and 60 healthy controls were enrolled. T_1_-maps (modified Look-Locker inversion recovery) and T_2_-maps (3 steady-state free-precession images with different preparation times) were obtained in a basal, mid-ventricular and apical short-axis slice. T_1_-maps were repeated after Gadobutrol i.v. T_1_- and T_2_-relaxation times and the partition coefficient were determined for every segment. Focal fibrosis was assessed with late enhancement images (LGE).

## Results

**Figure 1:**
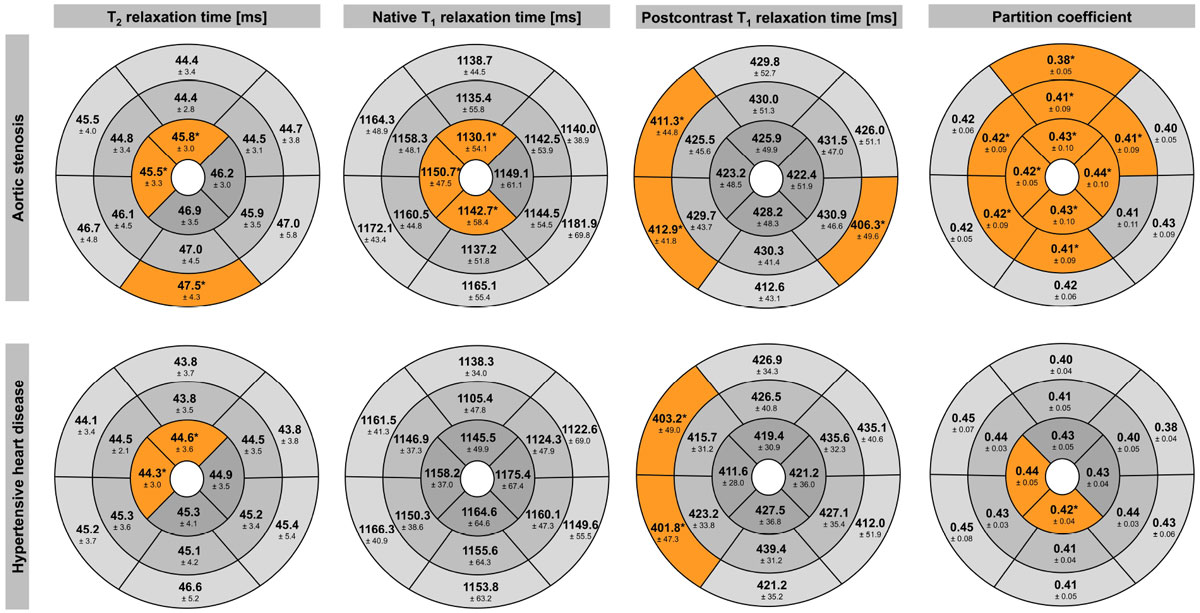
**Mean ± SD of T**_**2**_**- and T**_**1**_**-relaxation times and partition coefficient per segment**. Segments that differed significantly from healthy controls are marked "*" and highlighted in orange.

**Figure 2:**
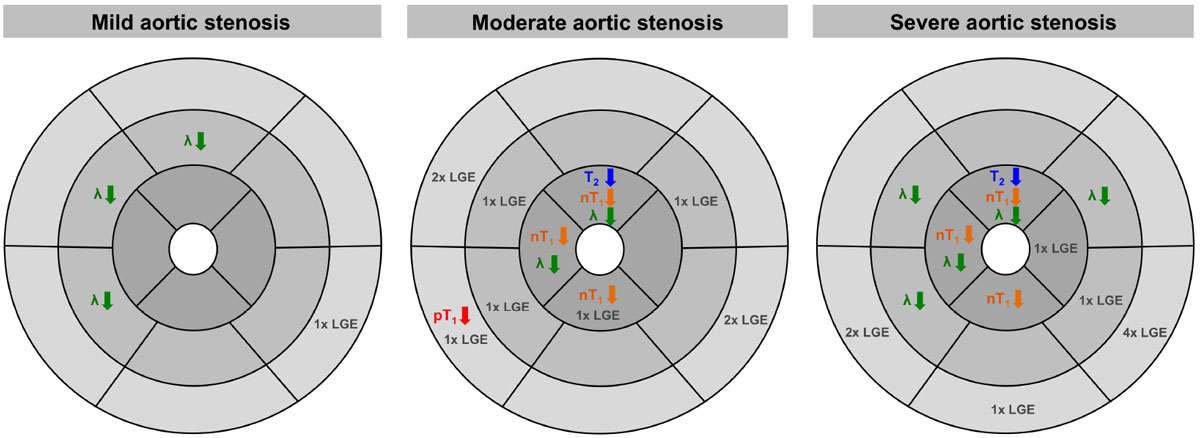
**Synopsis of abnormal findings when comparing each AS severity grade with healthy controls**.

Figure 1 shows the T_2_- and T_1_-relaxation times as well as the partition coefficient for each segment. Figure 2 provides a synopsis of the abnormal segments separated for AS severity grades. In AS, post-contrast T_1_-values were reduced at the base (septum, inferolateral) indicating fibrosis, in the septum even without the presence of LGE. Apical segments differed from controls by reduced T_2_- and native T_1_-values and partition coefficient, indicating abnormal regional tissue composition. With increasing AS severity, the number of segments with abnormal tissue composition increased. In HYP, post-contrast T_1_-values were abnormal in the basal septum indicating fibrosis, even without the presence of LGE. Partition coefficient and T_2_-relaxation times were reduced in the apex compared to controls.

## Conclusions

Severe AS, but also moderate AS and - to less extent - HYP exhibit abnormal regional tissue composition. Multiparametric segmental mapping has potential to detect organ damage at an early stage.

